# Evaluation and refinement of the PRESTARt tool for identifying 12–14 year olds at high lifetime risk of developing type 2 diabetes compared to a clinicians assessment of risk: a cross-sectional study

**DOI:** 10.1186/s12902-019-0410-3

**Published:** 2019-07-25

**Authors:** Laura J. Gray, Emer M. Brady, Olatz Albaina, Charlotte L. Edwardson, Deirdre Harrington, Kamlesh Khunti, Joanne Miksza, João Filipe Raposo, Ellesha Smith, Andriani Vazeou, Itziar Vergara, Susann Weihrauch-Blüher, Melanie J. Davies, on behalf of the PRE-STARt Collaborative, Melanie Davies, Kamlesh Khunti, Susan Enright, Emer Brady, Laura Gray, Charlotte Edwardson, Deirdre Harrington, Thomas Yates, Georgie Surridge, Joana Mora Amengual, Esteban de Manuel, Miren David Iturralde, Sara Ponce, Irati Erreguerena, Maider Mateo-Abad, Amaia Perales, Garbiñe Aizpuru, Susann Weihrauch-Blüher, Roland Pfaeffle, Peter Schwarz, Andrea Grimm, Rogerio Ribeiro, João Filipe Raposo, Andriani Vazeou, Thomai Karagiozoglou-Lampoudi, Vana Mitravela Maria Chatzipsalti, Evangelia Charmandari, Aggeliki Apostolou, Penio Kassari, Kalliopi Kouloufakou-Gratsia, Eirini Papadimitriou, Konstantinos Michalakis, Emma Wilmot, James Greening, Elena Alustiza, Irene Ozcoidi, Sagrario Fuentes, Sofia Vidal Castro, Rosa Maria Martins Pina, Andriani Vazeou, Susann Weihrauch-Blüher, Peter Schwarz, Prem Sundaram, Francesco Zaccardi, Elena Alustiza, Irene Ozcoidi, Sagrario Fuentes, Rosa Pina, Sofia Castro, Julia Galhardo, Christos Mantzoros, Andriani Vazeou, Susanna Wiegand

**Affiliations:** 1 Department of Health Sciences, College of Life Sciences, University of Leicester, George Davies Centre, University Road, Leicester, LE1 7RH UK; 2 Leicester Diabetes Centre, University Hospitals of Leicester, Leicester, LE5 4PW UK; 3 Kronikgune, Torre del BEC (Bilbao Exhibition Centre), Ronda de Azkue, 1, 48902 Barakaldo, Bizkaia Spain; 4 Diabetes Research Centre, University of Leicester, Leicester, LE5 4PW UK; 5 Associacao Protectora dos Diabeticas de Portugal, Lisboa, Portugal; 6 Diabetes Center, Department of Pediatrics, P&A Kyriakou Children’s Hospital, Athens, Greece; 7 Unidad de Investigación APOSIs Gipuzkoa, Osakidetza, Instituto Biodonostia, San Sebastián, Spain; 8 Red de Investigación en Servicios de Salud y Cronicidad REDISSEC, San Sebastián, Spain; 9 Integrated Research and Treatment Center (IFB) Adiposity Diseases, University of Leipzig, Leipzig, Germany; 10 Department of Pediatrics/ Pediatric Endorinology I, University Hospital of Halle/S, Halle, Germany

**Keywords:** Adolescent, Risk factors, Diabetes mellitus, type 2, Europe, Obesity

## Abstract

**Background:**

Traditionally Type 2 Diabetes Mellitus (T2DM) was associated with older age, but is now being increasingly diagnosed in younger populations due to the increasing prevalence of obesity and inactivity. We aimed to evaluate whether a tool developed for community use to identify adolescents at high lifetime risk of developing T2DM agreed with a risk assessment conducted by a clinician using data collected from five European countries. We also assessed whether the tool could be simplified.

**Methods:**

To evaluate the tool we collected data from 636 adolescents aged 12–14 years from five European countries. Each participant’s data were then assessed by two clinicians independently, who judged each participant to be at either low or high risk of developing T2DM in their lifetime. This was used as the gold standard to which the tool was evaluated and refined.

**Results:**

The refined tool categorised adolescents at high risk if they were overweight/obese and had at least one other risk factor (High waist circumference, family history of diabetes, parental obesity, not breast fed, high sugar intake, high screen time, low physical activity and low fruit and vegetable intake). Of those found to be at high risk by the clinicians, 93% were also deemed high risk by the tool. The specificity shows that 67% of those deemed at low risk by the clinicians were also found to be a low risk by the tool.

**Conclusions:**

We have evaluated a tool for identifying adolescents with risk factors associated with the development of T2DM in the future. Future work to externally validate the tool using prospective data including T2DM incidence is required.

**Electronic supplementary material:**

The online version of this article (10.1186/s12902-019-0410-3) contains supplementary material, which is available to authorized users.

## Background

In 2017, 425 million people had diabetes worldwide, this is projected to increase by 16% to 629 million by 2045 [1]. Around 90% of these are diagnosed with Type 2 Diabetes Mellitus (T2DM). Worldwide, 352 million people are at risk of developing T2DM and around 1 in 2 adults with T2DM are undiagnosed [1]. Historically T2DM was associated with older age, but over the past 15 years dramatic rises in T2DM are being seen in children, adolescents and young adults [2]. A study assessing the prevalence of T2DM in people aged 10–19 years in the United States found a 30% increase in prevalence between 2001 and 2009 [3]. Similar increases have been reported in the UK [4, 5]. Those with early onset T2DM (for example those diagnosed before 40 years old), seem to represent a high risk group, with long disease exposure leading to the early onset of microvascular and macrovascular complications [6]. Emerging evidence also suggests that early onset T2DM is associated with a more extreme phenotype than that seen in older adults [7]. Early onset has additional psycho-societal implications [8]. Those affected by early onset T2DM are of working age, studies show that diabetes is associated with a significant negative impact on the ability-to-work [8]. To date there is very little data regarding undiagnosed T2DM or the number of children, adolescents and young adults at risk of developing diabetes. One UK-based clinical trial recruited overweight and obese 18–40 year olds. Of the 193 participants recruited, 5% had undiagnosed T2DM and 18% had elevated glucose levels putting them at risk of developing T2DM [9].

Screening to identify adults at risk of developing T2DM is recommended by national bodies, such as NICE in England and Wales [10]. Such guidance usually recommends a two stage approach where a non-invasive risk score which assesses the presence of risk factors is used to pre-screen before a blood test is taken to assess HbA1c or glucose levels in those at high risk [10, 11]. Given T2DM can have a long asymptomatic phase, risk scores can also be used to identify people with undiagnosed T2DM. To date a plethora of risk scores have been developed and validated for use in adults to identify those at risk of either having undiagnosed T2DM or developing it in the future [12, 13]. Risk scores reduce the number of people requiring blood tests, help people understand their modifiable risk factors and have been shown to reduce the cost of screening and increase uptake [14, 15]. Identifying those at risk of developing T2DM and providing prevention programmes is very effective, with the landmark studies in the area showing a 58% reduction in the development of T2DM [16].

A collaboration between sites from five European Countries (Germany, Greece, Portugal, Spain and UK) developed the PRESTARt tool to identify adolescents (defined here as 12–14 year olds) with risk factors associated with the lifetime development of T2DM and to develop a prevention programme for high risk adolescents. The tool defined adolescents at high risk if they had both high levels of screen time and were overweight/obese and had one other of the included risk factors: high waist circumference; Acanthosis Nigricans; first degree family history of diabetes; non-Caucasian ethnicity; metabolic syndrome; rapid weight gain in 1st year; pre-diabetes; high sugar intake; fatty liver disease; parental obesity; polycystic ovary syndrome; small for gestational age; and not breast fed.

The aim of this study was to evaluate whether adolescents identified at high lifetime risk of T2DM by the PRESTARt tool agree with a risk assessment conducted by a clinician using data collected from sites in five European countries. We also assessed whether the tool could be simplified.

## Methods

We conducted a cross-sectional study of 12–14 years olds from sites in each of the five countries involved (Germany, Greece, Portugal, Spain and UK). These data were then assessed by a group of clinicians, who in their expert opinion deemed each participant to be at high or low risk of developing T2DM in their lifetime. This was used as the gold standard to which the tool was evaluated and refined. The cross-sectional study, outcome adjudication and evaluation of the tool are described in detail below.

### Cross-sectional study

We collected data from adolescents aged 12–14 years inclusive and their families from sites in five European countries. At each site local research ethics and regulatory approvals were obtained before recruitment commenced.

The inclusion and exclusion criteria were purposely broad. Only 12–14 year olds inclusive were included (as stipulated by the terms of the European Union tender) and those who were willing and able to give written informed assent (after obtained written informed parental/guardian consent). Individuals were ineligible if did not meet the inclusion criteria and/or had an existing diagnosis of type 1 or type 2 diabetes mellitus.

We planned to sample across the BMI distribution with over sampling at the higher BMI percentiles to ensure we recruited sufficient numbers of participants with risk factors for developing type 2 diabetes in order to be able assess the tool. The aim being to recruit between 10 and 25% with normal weight, between 25 and 50% overweight and between 30 and 50% obese as defined by the World Health Organisation (WHO) BMI for age reference charts for children aged 5–19 years [17]. The target sample size was 500 adolescents (100 per country). This minimum sample size was chosen as methodological studies have suggested that 100–200 cases and 100–200 non-cases should be included for the external validation of risk prediction models [18]. Using the proposed sampling frame, we estimated that at least 100 of the 500 adolescents recruited should be at high risk. The final sample size recruited was 636 adolescents. A variety of recruitment settings were used. In Spain, Greece and Germany potential participants were identified in clinical settings, whereas schools were used in Portugal and the UK.

An extensive data set from both the child and parents/guardians, covering health and family history, diet and lifestyle, anthropometric, puberty stage and biochemical measures were collected (described below). Standard operating procedures (SOP) for each of the measurements described were agreed and followed by each country, technicians collecting data were trained using these SOPs. Data were collected on standardised data collection forms and entered into web-based database developed by the Leicester Clinical Trials Unit.

Family history and current health status (see Additional file 1 for the data collected form used): The parents/guardians were asked about their family history of chronic disease such as T2DM, gestational diabetes, cardiovascular disease and stroke in themselves or their immediate relatives. Details of the child’s own birth and health history were reported including items such as child’s birth weight, their gestational period and whether they were breast or formula fed. Ethnicity was collected in Germany, Spain and UK only due to ethical requirements in Greece and Portugal.

Diet and lifestyle questionnaire (see additional file 2 for an example of the questionnaire used): A questionnaire booklet was collated to assess the child’s diet and lifestyle habits and included questions about risk factors that may have an association with chronic disease risk. This included the PACE+ questionnaire to assess physical activity levels [19]; The Adolescent Sedentary Activity Questionnaire to assess time spent sedentary [20]; and questions pertaining to frequency of breakfast consumptions, snacks, fruit and vegetables and sugary drink consumption [21–23].

Biological maturity status: The Tanner stage that the child had reached was self-reported using the Tanner scale pictures [24]. This questionnaire was not administered at the Portuguese site and was assessed by a paediatrician in Spain at the request of their ethics committees.

Anthropometric measurements: Weight was measured to the nearest 0.1 kg and height was measured to the nearest 0.1 cm using a clinically approved scale and a portable stadiometer, respectively. Body mass index (BMI) was calculated as weight (kg)/height (m)^2^ and was converted to a BMI percentile based on WHO growth charts [17]. Waist, neck and upper arm circumferences were measured with an inelastic anthropometry tape. Waist circumference was measured to the nearest 0.1 cm as the midpoint between the lower costal margin and iliac crest. Neck and upper arm circumferences were also measured to the nearest 0.1 cm at the appropriate anatomical locations. For example, upper arm circumference was measured at the mid-point on the belly of the bicep muscle (i.e. highest point). Neck circumference was measured at the mid-point of the neck. Arterial blood pressure was measured using an automated sphygmomanometer with an appropriate sized cuff while the participant was seated, and having rested quietly for 5 minutes. Three measurements were obtained for blood pressure and the average of the last two used for analysis.

Biochemical measures: Triglycerides, glucose, high density lipoprotein cholesterol (HDL-C) and total cholesterol were measured using a point-of-care testing (POCT) device (CardioChek® system) and low density lipoprotein cholesterol (LDL-C) automatically calculated. Capillary blood samples (15 to 40 μL) were taken from each participant using the finger prick method. The CardioChek® system is certified by the Cholesterol Reference Method Laboratory Network (CRMLN) and National Cholesterol Education Program (NCEP), is FDA-cleared, CE-marked, internationally registered, and is CLIA-waived by the Centers for Medicare & Medicaid Services, USA. HbA1c was measured using POCT with the A1C Now®^+^ system, BHR Pharmaceuticals Limited (UK). The A1CNow + system is annually certified by the National Glycohemoglobin Standardization Program (NGSP). Having successfully completed rigorous testing requirements, the A1CNow + system was awarded a Certification of Traceability to the Diabetes Control and Complications Trial (DCCT) Reference Method (http://www.ngsp.org/bground.asp). Participants were not specifically required to fast for these blood tests; time of the last meal consumed or whether they were fasting was recorded.

### Evaluation of the tool

The development of the PRESTARt tool has been described previously [25]. Briefly, given there were no data available to develop such a tool a novel approach was used. The American Diabetes Association (ADA) diabetes screening recommendations for children and young people [26] and the results from a systematic review assessing predictors of diabetes risk in children and adolescents were used to develop the tool. Once a pool of potential risk factors for inclusion were identified, a Delphi study was undertaken to decide which of these should be included in the tool [25].

To assess the performance of the PRESTARt tool, the lifetime risk status of each participant from the cross-sectional study needed to be established and then compared to the result of the tool. A pool of clinicians, with two allocated to each participant, independently judged each participant’s lifetime risk status using the extensive data collected during the cross-sectional study. Where the clinical assessments did not agree a third clinician adjudicated. Each country provided a pool of three clinical assessors. A bespoke database was developed which presented each participants anonymised data in an easy to read and accessible format and then the last page of the system recorded the assessor’s outcome. Missing data were shown as blank responses and therefore the reviewers could not use this in their assessment. Initially all of the clinicians assessed the same 20 participants to train them in using the system and the process. Feedback was provided after this training exercise. This was followed by two rounds of assessment, one when half the data had been collected and cleaned and one at the end of the study. Assessors were randomised to participants in country based clusters – the idea being that for difficult cases the three assessors could discuss the case, although this was not required.

The PRESTARt tool, as previously described, gives a binary outcome of either low or high risk. Participants were defined at high lifetime risk if they had both high levels of screen time (≥2 h of TV/computer viewing per day) and were overweight/obese (≥85th BMI percentile) and had one other of the included risk factors (high waist circumference (defined using the following age/sex cut points: 12 male: 84.5 cm; 12 female: 81.2 cm; 13 male: 87.9 cm; 13 female: 84.1 com; 14 male: 91.3 cm; 14 female: 86.9 cm), acanthosis nigricans, first degree family history of diabetes, non-Caucasian ethnicity, metabolic syndrome (defined as having three or more of: (i) high blood pressure; (ii) high cholesterol; (iii) high triglycerides; (iv) high blood glucose levels, but not in the diabetes range), rapid weight gain in 1st year (≥2 lb. (908 g) a month), pre-diabetes, high sugar intake (≥1.5 cans (or 532mls) of carbonated sugar sweetened beverages/fruit juice a day), fatty liver disease, parental obesity (BMI ≥30 kg/m^2^ if White European or 27 kg/m^2^ for other ethnicities), polycystic ovary syndrome, small for gestational age (using published guidelines [27]), and not breast fed).

The tool outcome was compared to the adjudicated outcome using sensitivity, specificity, positive predictive value (PPV), negative predictive value (NPV) and the area under the receiver operating curve (ROC) value.

### Refinement of the tool

To assess if the tool developed could be simplified to reduce the burden on the completer, without losing statistical performance (in comparison to the clinicians assessment of risk), we assessed the effect on the statistical measures of performance (using area under the receiver operating curve, sensitivity, specificity, PPV and NPV) of removing each of the risk factors included. We also sought opinions from the PRE-STARt Collaborative on ways to simplify and/or amend the tool. These included suggestions from the members about other modifiable risk factors that could be important for improving the healthy lifestyle messages provided by the tool. An example of such an approach is the FINDRISC diabetes score for use in adults. This score includes questions asking about physical activity and fruit and vegetable intake [28], not because these improve the statistical performance of the score but because they are modifiable risk factors and are therefore there for education purposes. A risk score which contains only non-modifiable risk factors may give the impression to completers that their risk cannot be changed. The evaluation was repeated for the final tool.

## Results

### Cross-sectional study

In total 636 participants were recruited into the study (Greece 100 (15.7%), Germany 100 (15.7%), Portugal 226 (35.5%), Spain 129 (20.3%), and UK 81 (12.7%)). Of these, 52.2% were male, with a mean age of 13.3 years. The full results are given in Table 1 and Additional file 3: Tables S1-S7. There was large variation in BMI in those recruited between countries, overall 56% of participants had BMI over the 85th percentile, but this ranges from 32% of those recruited in Portugal up to 91% of those recruited in Spain. Although we aimed to quota recruit by BMI this was not possible in all countries. The majority of participants were Caucasian from the sites in Germany and Spain. In the UK site 54% were of non-white ethnicity, reflecting the ethnic diversity of the area where recruitment took place. We were unable to collect ethnicity data in Greece and Portugal. In terms of cardiovascular risk factors, 29 participants (5%) had high blood pressure, 15 (3%) had HbA1c over 6.0% which would be deemed high risk in adults, with five (1%) having an HbA1c over 6.5%, i.e. indicative of undiagnosed T2DM. In terms of high cholesterol, four (1%) participants had high total cholesterol and 11 (2%) had high LDL cholesterol.

**Table 1:** Characteristics of the sample used for evaluation of the tool overall and by country. Data given as mean (SD) unless otherwise stated

	UK	Germany	Portugal	Spain	Greece	Total
Number recruited (%)	81 (12.7)	100 (15.7)	226 (35.5)	129 (20.3)	100 (15.7)	636 (100.0)
Sex, male (*n* (%))	60 (74.1)	50 (50.0)	106 (46.9)	60 (46.5)	56 (56.0)	332 (52.2)
Age	13.5 (0.8)	13.3 (0.8)	13.5 (0.8)	12.9 (0.6)	12.9 (0.6)	13.3 (0.8)
Ethnicity (*n* (%))						
Caucasian	37 (45.7)	92 (92.0)	–	111 (86.1)	–	240 (37.9)
Other	44 (54.3)	8 (8.0)	–	18 (14.0)	–	70 (11.0)
Missing	0 (0.0)	0 (0.0)	226 (100.0)	0 (0.0)	100 (100.0)	326 (51.3)
BMI	20.5 (4.4)	24.9 (6.4)	20.9 (3.8)	25.6 (4.0)	25.3 (5.6)	23.1 (5.2)
>85th Percentile^17^ (*n* (%))	28 (34.6)	65 (65.0)	73 (32.3)	117 (90.7)	71 (71.0)	354 (55.7)
Waist (cm)^5^	74.9 (12.1)	80.9 (15.9)	75.0 (10.1)	84.6 (11.3)	84.8 (14.3)	79.4 (13.1)
Neck (cm)	32.7 (2.9)	33.6 (3.4)	31.6 (2.9)	33.8 (2.8)	34.9 (3.1)	33.0 (3.2)
Upper arm (cm)^6^	25.6 (3.8)	27.8 (5.1)	26.1 (3.3)	29.7 (3.4)	28.9 (4.7)	27.4 (4.2)
Body fat (%)^7^	19.7 (10.0)	29.9 (13.5)	22.4 (8.9)	–	29.6 (9.2)	24.9 (10.9)
Fat-free mass (Kg)	42.3 (8.2)	45.3 (9.2)	–	–	44.9 (7.4)	44.2 (8.3)
Muscle mass^8^ (Kg)	40.1 (7.8)	–	35.3 (3.8)	–	43.8 (7.1)	37.9 (6.5)
Systolic BP^9^ (mmHg)	110.4 (10.5)	115.6 (10.2)	103.7 (12.5)	113.9 (12.2)	113.2 (10.8)	109.9 (12.6)
Diastolic BP (mmHg)	65.3 (8.6)	70.8 (8.1)	66.4 (8.5)	66.6 (8.6)	67.0 (8.4)	67.1 (8.6)
BP high risk^2^ (*n* (%))	1 (1.23)	8 (8.0)	9 (3.9)	7 (5.43)	4 (4.0)	29 (4.6)
Heart rate^10^(bpm)	77.2 (11.0)	81.7 (11.4)	82.3 (14.0)	77.7 (13.4)	79.4 (12.3)	80.0 (13.2)
HbA1c^11^ (%)	5.5 (0.7)	5.2 (0.3)	5.3 (0.4)	5.4 (0.3)	5.2 (0.4)	5.3 (0.4)
≥6.0% (*n* (%))	5 (8.3)	0 (0.0)	8 (3.6)	1 (0.8)	1 (1.0)	15 (2.5)
≥6.5% ^1^(*n* (%))	1 (1.7)	0 (0.0)	3 (1.4)	0 (0.0)	1 (1.0)	5 (0.8)
Glucose (mmol/L)^13^	4.9 (0.8)	4.8 (0.5)	5.1 (0.7)	4.6 (0.7)	4.4 (0.7)	4.8 (0.7)
High risk glucose^3^ (*n* (%))	0 (0.0)	0 (0.0)	1 (0.4)	1 (0.8)	1 (1.0)	3 (0.5)
Triglycerides (mmol/L)^14^	1.2 (1.4)	1.1 (0.7)	1.1 (0.8)	0.9 (0.4)	1.0 (0.6)	1.1 (0.8)
Total cholesterol (mmol/L)^12^	3.4 (1.2)	3.5 (0.8)	3.4 (0.6)	3.5 (0.7)	3.5 (1.0)	3.5 (0.8)
≥6.0 mmol/L ^1^(*n* (%))	1 (1.2)	1 (1.0)	0 (0.0)	0 (0.0)	2 (2.0)	4 (0.6)
HDL cholesterol (mmol/L)^15^	1.4 (0.4)	1.2 (0.3)	1.3 (0.3)	1.2 (0.4)	1.3 (0.4)	1.3 (0.4)
LDL cholesterol (mmol/L)^16^	1.6 (0.5)	2.2 (0.7)	1.7 (0.4)	1.9 (0.1)	1.9 (0.6)	1.8 (0.5)
≥ 3.0 mmol/L^1^ (*n* (%))	0 (0.0)	8 (11.6)	1 (0.4)	0 (0.0)	2 (2.0)	11 (1.7)

^1^Proportion of participants that are considered to be at high risk. ^2^High risk blood pressure is those that have average systolic BP ≥ 120 and average diastolic BP ≥ 80 (red zone). ^3^Fasting participants are considered to be at high risk (red zone) when glucose ≥ 7.0 mmol/L and non-fasting participants are considered to be at high risk when glucose level ≥ 11.1 mmol/L. ^5^1 missing value, ^6^10 missing values, ^7^135 missing values, ^8^2 missing values ^9^9 missing values, ^10^78 missing values, ^11^34 missing values, ^12^43 missing values, ^13^9 missing values, ^14^50 missing values, ^15^46 missing values, ^16^285 missing values, ^17^85th percentile equivalent to z-score of 1.04

When applying the PRESTARt tool to the cross-sectional study data, 214 (33.7%) participants were found to be at high risk. 241 (37.9%) participants were defined at high risk by the clinical review (Table 2). For 76% of cases the two clinical experts agreed, therefore 24% required a third party to reach a final decision on the risk status. Both when using the tool and the clinical assessment there were differences between countries in terms of the percentage of participants at high risk. This reflects the differences in participant characteristics seen between the countries.

**Table 2:** Comparison of the results of the clinician review and the original and refined PRESTARt tools both by country and overall. Data given as n (%)

	UK	Germany	Portugal	Spain	Greece	Total
High risk, clinician	27 (33.3)	53 (53.0)	43 (19.0)	67 (51.9)	51 (51.0)	241 (37.9)
High risk, original tool	18 (22.2)	51 (51.0)	30 (13.3)	51 (39.5)	64 (64.0)	214 (33.7)
High risk, refined tool	26 (32.1)	63 (63.0)	69 (30.5)	116 (89.9)	69 (69.0)	343 (53.9)

### Evaluation of the PRESTARt tool

Table 3 shows the statistical performance of the tool. Overall 64% of those assessed to be at high risk by the clinicians were also found to be high risk when using the tool (sensitivity). Conversely, of the 214 who were found to be at high risk by the tool 72% of these were also found to be at high risk by the clinicians (PPV). The specificity and NPV look at those found to be low risk and agreement within this group. Eighty five percent of those deemed to be low risk by the clinicians were also found to be low risk when using the tool (specificity). Of those with a low risk from the tool, 79% were also found to be low risk by the clinicians (NPV). Eighteen participants (6.41%) were deemed to be high risk by the clinicians were not overweight/obese. The area under the ROC was 0.74 (95% CI 0.71, 0.78) before refinement. In our data this value represents the probability that a randomly selected high risk adolescent will have a higher test result than a randomly selected low risk adolescent.

**Table 3:** Statistical performance of the initial and refined PRESTARt tool compared to a clinicians assessment of lifetime risk of T2DM

	Initial tool		Refined tool	
	%	95% CI	%	95% CI
Sensitivity	63.9	57.5, 70.0	92.5	88.5, 95.5
Specificity	84.8	80.9, 88.2	66.8	62.0, 71.5
Positive predictive value	72.0	65.4, 77.9	63.0	57.7, 68.0
Negative predictive value	79.4	75.2, 83.1	93.6	90.1, 96.2

### Refinement of the PRESTARt tool

The refinement of the PRESTARt tool was conducted in two stages, first assessing the effect of removing risk factors on the statistical performance and secondly incorporating requests from the PRE-STARt Collaborative and again assessing the effect of these on performance. Table 4 shows the area under the ROC curve for each of the tools assessed. When removing variables the performance of the tool remained fairly consistent (tools 1–12) until the removal of the rapid weight gain between 0 and 4 months, when the area under the ROC reduced to 0.73. The results of these analyses were presented to the members of the study steering committee. Members requested testing the following changes to the tool:

- Removal of high screen time from the core risk factors (Tool 13). Although sedentary behaviour has been associated with adult diabetes [29] this is an emerging area of research in those under 18 and members felt it is too premature to have screen time as a core risk factor ahead of physical inactivity. Meeting notes from stakeholder events suggested that this behaviour is one of the key modifiable behaviours that GPs report as a “problem” and for this reason could be a useful starting point in getting parents to think about their child’s lifestyle.
- Adding back in the questions about parental obesity and breast feeding (Tool 14). As these questions are relatively straight forward for parents to complete and are somewhat representative of the family lifestyle it was felt these risk factors would broaden the message around modifiable risk factors out to the wider family.
- Removal of the rapid weight gain question as parents involved in the study reported difficulties in understanding what this meant and actually remembering this detail (Tool 15).
- Adding back in the family history question but including 2nd degree as well as 1st degree, given the age of the participants the parents may not yet have developed diabetes (Tool 16).
- Adding in additional modifiable risk factors. We assessed adding the following modifiable risk factors to the score - high sugar intake, high screen time, low physical activity (< 60 mins per day), low fruit and vegetable intake (< 5 portions per day) (Tools 17–20).

**Table 4:** The process of refinement of the tool and the performance (measured using area under the receiver operating curve (AUROC)) of each version compared to a clinicians assessment of lifetime risk of T2DM

	Risk factors Core	Risk factors Plus one	AUROC
Tool	OO, ST	WC, AN, FLD, PCOS, PDM, MS, FH, ETH, WG04, SI, SGA, BF, OP	0.74 (0.71, 0.78)
Removing variables			
Tool 1	OO, ST	- AN	0.74 (0.71, 0.78)
Tool 2	OO, ST	- AN, FLD	0.74 (0.71, 0.78)
Tool 3	OO, ST	- AN, FLD, PCOS	0.74 (0.71, 0.78)
Tool 4	OO, ST	- AN, FLD, PCOS, SI	0.74 (0.71, 0.78)
Tool 5	OO, ST	- AN, FLD, PCOS, SI, MS	0.74 (0.71, 0.78)
Tool 6	OO, ST	- AN, FLD, PCOS, SI, MS, FH	0.74 (0.71, 0.78)
Tool 7	OO, ST	- AN, FLD, PCOS, SI, MS, FH, PDM	0.74 (0.71, 0.78)
Tool 8	OO, ST	- AN, FLD, PCOS, SI, MS, FH, PDM, ETH	0.75 (0.71, 0.78)
Tool 9	OO, ST	- AN, FLD, PCOS, SI, MS, FH, PDM, ETH, OP	0.75 (0.71, 0.78)
Tool 10	OO, ST	- AN, FLD, PCOS, SI, MS, FH, PDM, ETH, OP, BF	0.74 (0.71, 0.78)
Tool 11	OO, ST	- AN, FLD, PCOS, SI, MS, FH, PDM, ETH, OP, BF, SGA	0.74 (0.71, 0.77)
Tool 12	OO, ST	- AN, FLD, PCOS, SI, MS, FH, PDM, ETH, OP, BF, SGA, WG04	0.73 (0.69, 0.76)
Collaboration requested refinements			
Tool 13	OO	**-** AN, FLD, PCOS, SI, MS, FH, PDM, ETH, OP, BF, SGA	0.80 (0.76, 0.83)
Tool 14	OO	Tool 13 + OP, BF	0.81 (0.78, 0.84)
Tool 15	OO	Tool 14 – WG04	0.81 (0.78, 0.84)
Tool 16	OO	Tool 15 + FH or 2nd degree family history diabetes (FH2)	0.81 (0.78, 0.83)
Tool 17	OO	Tool 16 + SI	0.80 (0.77, 0.83)
Tool 18	OO	Tool 17 + ST	0.80 (0.78, 0.83)
Tool 19	OO	Tool 18 + low physical activity	0.80 (0.78, 0.83)
Tool 20 (final tool)	OO	Tool 19 + low fruit and veg	0.80 (0.77, 0.83)
Tool 21	OO	Tool 20 + AN	0.80 (0.77, 0.83)

*ST* Screen time, *OO* Overweight/obese, *WC* Waist circumference, *AN* Acanthosis nigricans, *FLD* Fatty liver disease, *PCOS* Polycystic ovary syndrome, *PDM* Pre-diabetes, *MS* Metabolic syndrome, *FH* Family history of diabetes, *ETH* Non-caucasian ethnicity, *WG04* Weight gain between 0 and 4 months old, *SI* Sugar intake, *SGA* Small for gestational age, *BF* Never fed breastmilk, *OP* Obese parent

All of the suggestions given above either improved the statistical performance of the tool or did not reduce it and where therefore incorporated into the final tool. The final tool is shown in additional file 4 and the number and percentage of participants with each risk factor in Table 5. The statistical measures from the evaluation of the refined tool are given in Table 3. The performance of the updated tool improved significantly. Of those found to be high risk by the clinicians, 93% are also deemed high risk by the tool. The specificity shows that 67% of those deemed at low risk by the clinicians are also found to be a low risk when using the tool. The NPV show that those receiving a low risk result from the tool are not being falsely reassured, as 94% of those with a low tool result were deemed to be low risk by the clinicians.

**Table 5:** The number and percentage of participants with each of the risk factors included in the refined PRESTARt tool

Risk factor	Number	Percentage
Body mass index (BMI) above the 85th percentile	355	55.8
High waist circumference	200	31.5
Watch TV/play computer games for more than 2 h a day	351	55.2
Do less than 60 min of physical activity a day	43	6.8
Eat less than 5 portions of fruit or vegetables a day	348	55.2
Have a family history of diabetes	73	11.5
Have a high sugar intake	89	14.0
Never fed on breast milk	112	17.6
Either parent/guardian obese	219	34.4

## Discussion

We have collected data from over 600 adolescents from five European countries. These data have been used to evaluate and refine the PRESTARt tool for identifying adolescents with risk factors for the development of T2DM in their lifetime. We have shown that the final refined tool performs well when compared to a clinician’s assessment of risk.

We have taken a novel and pragmatic approach to both the development and evaluation of this tool. The standard way of developing such a tool or score would be to use existing data to model the associations between risk factors and the outcome of interest. In this case we would need data which followed up a cohort of adolescents for decades so that the relationship between risk factors present in adolescence and the development of T2DM could be assessed. No such data were available and therefore a novel and pragmatic approach was taken. The tool was developed using the results from a systematic review and consensus study which identified risk factors for inclusion [25]. The tool assesses the presence of these risk factors rather than attributing weight to them, this was based on the format used for the ADA screening guidelines [26]. This approach gives equal weighting to all of the risk factors included, which although may not be appropriate, the performance of the tool shows that the outcome of the tool is usually in agreement with that of the clinicians. This also means that the tool is easy to use in practice as no calculations or specialist equipment are required. Therefore this tool could be completed by parents outside of a health care setting. Prior to implementation in clinical practice, all such tools should be validated [30]. To validate this tool a similar data set to that required for development would be needed, i.e. a longitudinal data set which follows up a representative sample of adolescents for many decades which records whether they develop T2DM. Given this was not available we have taken a different approach. We have evaluation the tool against a surrogate marker of diabetes risk, in this case clinical opinion. All participants were assessed by two clinicians independently, with a third being used where consensus was not found. In the majority of cases the two clinicians agreed. Therefore although this approach is not without its limitations, we believe a tool evaluated in this way does still does provide useful information and can be used to identify adolescents at risk without the input from clinicians, for example in a community setting. Ideally long term follow up of the cohort would allow validation against the development of T2DM to be undertaken. In the shorter term, additional validation of the final refined tool using other cross-sectional data is warranted, which could include extending the validated age range beyond 12–14 year olds.

We believe this is the first such tool developed for use in this age group in a European setting. Many risk tools/scores have been developed to assess diabetes risk in adults [13, 31], some of which have been validated in young adults (18–25 years) [32]. There are a number of notable differences between those tools developed for use in adults and the tool developed here. Firstly the sensitivity, the percentage of those with a high risk outcome also being assessed as high risk by the clinicians, is significantly higher than those seen for adult tools (92.5% compared to 70–80%) [13, 33]. High sensitivity can be due to the proportion being defined at high risk, i.e. a tool with 100% sensitivity may have defined the whole population at high risk. That is not the case here, 56% of those screened are defined at high risk by the refined tool, and this is in line with the tools developed for use in adults [34]. This may also reflect that the clinicians assessing the participants used weight as the primary driver for their assessment, this is also the primary risk factor in the tool as to be at high risk completers have to be overweight/obese with one or more additional risk factors. One could argue that weight alone should therefore be used to assess risk and indeed doing this does not hamper the performance of the tool (ROC 0.80, 95% CI 0.77, 0.82), however we believe a more holistic assessment of risk is important for a number of reasons. Firstly it makes the completer aware of a number of modifiable risk factors (screen time, physical activity, sugary drinks etc.), allowing individuals to target multiple risk factors. We also include family based risk factors, such as parental obesity, this may encourage family wide improvements in lifestyle – which are important in this group. Additionally there maybe stigma around having and/or being an overweight/obese child [35], making the tool less obesity orientated and more focussed on being healthy may reduce this. As previously discussed, this tool uses a pragmatic approach and does not assign weights to risk factors. This crude scoring may have also influenced the performance of the tool. Finally, many of the risk factors included in this tool are not included in the tools developed for use in adults [13]. Again this emphasises the needs for tailored risk identifications and prevention approaches.

This tool could be used to identify participants for lifestyle modification programmes aimed at diabetes prevention. Such programmes are usually only offered to adults with elevated glucose levels putting them at high risk of diabetes. Studies show that the prevalence of T2DM at earlier ages from childhood through to young adults is increasing [3–5] and therefore to be effective, prevention initiatives need to target younger age groups and/or provide family based approaches. This paper describes the first stage of a programme of funding, the second stage is to develop and evaluate a family based healthy living intervention. Described elsewhere, this evaluation shows that positive changes in health behaviours are associated with attendance at such programmes [36]. Therefore, this programme of research has shown that it is feasible to identify high risk adolescents using a tool and that healthy behaviour can be promoted through family based prevention workshops.

The data collected in this study has the potential to be used for further hypothesis generating cross-sectional research. The data set also highlights the risk profile of those included, adding to the growing evidence base for T2DM risk in young people in European countries.

The strengths of this study include having recruited over the planned sample size and the use of standardised data collection methods across five countries. Although the non-standard methodology used for developing, evaluating and refining the tool could be seen as a limitation, it could be used to inform the development of screening tools for other areas where no data on which to develop a tool exist, one such example maybe the development of screening tools for use in developing countries. The initial protocol for this study set out to purposely sample individuals to get a specific BMI distribution for the cohort, in practice this was not possible, which has led to differences between the countries included in terms of the cohort characteristics. Also we cannot guarantee the representativeness of the included samples within each country as the study was not designed to recruit representative samples. This is an important limitation which must be taken into account when using this data. Another important limitation is the restricted age range included within this study – 12-14 year olds only. This reflects the requirement from the funders of this work, future research should assess the validity of this tool in a wider age range. Ideally further validation would be undertaken in a population based cohort. In terms of the clinical review, a strength of this is the use of two independent reviewers for each individual. Unfortunately we did not collect data on how the clinicians made their decisions, which data they used to form these decisions and timeframe for developing T2DM used. These data could have informed which risk factors to include in the refined tool. Even though this was not conducted the final tool still maintain a high level of performance. Future work could conduct a qualitative study to establish how clinicians make decisions about future risk in adolescents. Adjudication was also performed within countries, i.e. the clinicians reviewing each participant all came from the same country and therefore we cannot assess between country differences in adjudication. The methods used for the refinement of the tool may also have affected the final tool developed. The refinement was completed in two stages, firstly we removed risk factors one at a time to try and simplify the tool while maintaining adequate performance. The order in which risk factors were removed was arbitrary and this could have affected the final tool produced. Although the second stage of the refinement was based on stakeholder requests and here risk factors were removed/included based on clinical opinion and taking into account the ease of completion when used in practice. Therefore we feel that although the final tool produced is pragmatic the high levels of performance are reassuring.

## Conclusions

In conclusion, we have refined and evaluated a tool for identifying adolescents with risk factors associated with the lifetime development of T2DM. This tool has high agreement with clinical opinion. Future work to validate the tool using prospective data is required. The PRESTARt tool could be used both by parents and health care professionals to identify adolescents for referral into diabetes prevention programmes.

## Additional files

Additional file 1: This file includes the Case report form which was used to capture data from participants. (PDF 1100 kb)

Additional file 2: This file includes an example (female participant version) of the questionnaire completed by all participants. (PDF 400 kb)

Additional file 3: **Table S1.** Family medical history summary statistics. Data reported as n (%). **Table S2.** Participant medical history summary statistics. Data reported as n (%) unless otherwise stated. **Table S3**. Socio-economic summary statistics. Data reported as n (%). **Table S4.** Perinatal history summary statistics. Data reported as mean (sd) unless otherwise stated. **Table S5.** Summary statistics for participant’s physical activity and sedentary behaviour. Data reported as n (%) unless otherwise stated. **Table S6.** Summary statistics for participant’s diet. Data reported as mean (sd). **Table S7.** Tanner stage summary statistics. Data reported as n (%). (DOCX 29 kb)

Additional file 4: This file includes the final prestart tool. (DOCX 13 kb)

## Data Availability

The datasets used and/or analysed during the current study are available from the corresponding author on reasonable request.

## Abbreviations

ADA: American Diabetes Association
BMI: Body Mass Index
CRMLN: Cholesterol Reference Method Laboratory Network
DCCT: Diabetes Control and Complications Trial
HDL-C: High density lipoprotein cholesterol
LDL-C: Low density lipoprotein cholesterol
NCEP: National Cholesterol Education Program
NGSP: National Glycohemoglobin Standardization Program
NPV: Negative predictive value
POCT: Point-of-care testing
PPV: Positive predictive value
ROC: Receiver operating curve
SOP: Standard operating procedures
T2DM: Type 2 Diabetes Mellitus
WHO: World Health Organisation
