# Supplementary material for: Evaluation and refinement of the PRESTARt tool for identifying 12–14 year olds at high lifetime risk of developing type 2 diabetes compared to a clinicians assessment of risk: a cross-sectional study
Source: BMC Endocr Disord. 2019 Jul 25;19:79. doi: 10.1186/s12902-019-0410-3 (PMC6659313; doi:10.1186/s12902-019-0410-3)
Supplement: Supplementary file 1 — This file includes the Case report form which was used to capture data from participants. (PDF 1100 kb) [file 12902_2019_410_MOESM1_ESM.pdf]

PRE-STARt : Phenotyping Study

Case Report Form (Version 1 10.06.2015)

Participant Study ID: PHEN – X – 000	Date of Clinic Visit: DD – MM – YYYY

PERSONAL DETAILS CHECK

Are the contact details correct?

Yes ☐

No ☐ - please check that the correct participant file is being used before completing a new contact sheet

Gender of the participant: Male ☐ Female ☐

Date of birth of the participant: DD - MM - YYYY Age: YY - MM

INCLUSION CRITERIA – An answer of **YES** must be given to all of the following questions for the participant to be eligible.

Do they have a good understanding of written and verbal English?	Y	N
Has their parent/guardian provided consent?	Y	N
Has the child provided assent?	Y	N
Are they aged between 12 and 14 years old (inclusive)?	Y	N

EXCLUSION CRITERIA – An answer of **NO** must be given to the following for the participant to be eligible.

Does the child have existing Type 1 or Type 2 Diabetes Mellitus	Y	N

ETHNICITY – Please tick the box that best describes the participant's and parents' ethnic origin.

	Child	Mother	Father
White			
White British	☐	☐	☐
White Irish	☐	☐	☐
Other White background	☐	☐	☐
Mixed			
White and Black Caribbean	☐	☐	☐
White and Black African	☐	☐	☐
White and Asian	☐	☐	☐
Any other mixed race	☐	☐	☐
Asian or Asian British			
Indian	☐	☐	☐
Pakistani	☐	☐	☐
Bangladeshi	☐	☐	☐
Any other Asian background	☐	☐	☐
Black or Black British			
Caribbean	☐	☐	☐
African	☐	☐	☐
Any other Black background	☐	☐	☐
Chinese			
Chinese	☐	☐	☐
Other			
Other Ethnic group	☐	☐	☐
Specify			

What your relation is to the participant?

Mother ☐ Father ☐ Aunt ☐ Uncle☐ Grandmother☐ Grandfather ☐ Brother ☐ Sister☐ Non-biologically related guardian ☐

Other please specify ________________________

FAMILY HISTORY – Please indicate whether family member(s) of the participant (young person) have had a physician diagnosis of the following conditions. Please indicate the age at which they first presented with the condition(s). Please tick 'Unk' for cases where the presence of a condition is not known and write 'Unk' next to Age when the age of onset is not known.

	Heart Disease	High Cholesterol	Cardiovascular Disease	Type 1 Diabetes	Type 2 Diabetes	Other (please give details)
Maternal Grandfather	Y □ N □<br>Unk □<br>Age _______	Y □ N □<br>Unk □<br>Age _______	Y □ N □<br>Unk □<br>Age _______	Y □ N □<br>Unk □<br>Age _______	Y □ N □<br>Unk □<br>Age _______	Y □ ____________________________________<br>N □ Unk □<br>Age _______
Maternal Grandmother	Y □ N □<br>Unk □<br>Age _______	Y □ N □<br>Unk □<br>Age _______	Y □ N □<br>Unk □<br>Age _______	Y □ N □<br>Unk □<br>Age _______	Y □ N □<br>Unk □<br>Age _______	Y □ ____________________________________<br>N □ Unk □<br>Age _______
Paternal Grandfather	Y □ N □<br>Unk □<br>Age _______	Y □ N □<br>Unk □<br>Age _______	Y □ N □<br>Unk □<br>Age _______	Y □ N □<br>Unk □<br>Age _______	Y □ N □<br>Unk □<br>Age _______	Y □ ____________________________________<br>N □ Unk □<br>Age _______
Paternal Grandmother	Y □ N □<br>Unk □<br>Age _______	Y □ N □<br>Unk □<br>Age _______	Y □ N □<br>Unk □<br>Age _______	Y □ N □<br>Unk □<br>Age _______	Y □ N □<br>Unk □<br>Age _______	Y □ ____________________________________<br>N □ Unk □<br>Age _______
Mother	Y □ N □<br>Unk □<br>Age _______	Y □ N □<br>Unk □<br>Age _______	Y □ N □<br>Unk □<br>Age _______	Y □ N □<br>Unk □<br>Age _______	Y □ N □<br>Unk □<br>Age _______	Y □ ____________________________________<br>N □ Unk □<br>Age _______
Father	Y □ N □<br>Unk □<br>Age _______	Y □ N □<br>Unk □<br>Age _______	Y □ N □<br>Unk □<br>Age _______	Y □ N □<br>Unk □<br>Age _______	Y □ N □<br>Unk □<br>Age _______	Y □ ____________________________________<br>N □ Unk □<br>Age _______

FAMILY HISTORY (CONTINUED) – Please indicate whether family member(s) of the participant (young person) have had a physician diagnosis of the following conditions. Please indicate the age at which they first presented with the condition(s). Please tick 'Unk' for cases where the presence of a condition is not known and write 'Unk' next to Age when the age of onset is not known.

	Heart Disease	High Cholesterol	Cardiovascular Disease	Type 1 Diabetes	Type 2 Diabetes	Other (please give details)
Sibling 1<br>N/A □	Y □ N □<br>Unk □<br>Age ______	Y □ N □<br>Unk □<br>Age ______	Y □ N □<br>Unk □<br>Age ______	Y □ N □<br>Unk □<br>Age ______	Y □ N □<br>Unk □<br>Age ______	Y □ ________________________________<br>N □ Unk □<br>Age ______
Sibling 2<br>N/A □	Y □ N □<br>Unk □<br>Age ______	Y □ N □<br>Unk □<br>Age ______	Y □ N □<br>Unk □<br>Age ______	Y □ N □<br>Unk □<br>Age ______	Y □ N □<br>Unk □<br>Age ______	Y □ ________________________________<br>N □ Unk □<br>Age ______
Sibling 3<br>N/A □	Y □ N □<br>Unk □<br>Age ______	Y □ N □<br>Unk □<br>Age ______	Y □ N □<br>Unk □<br>Age ______	Y □ N □<br>Unk □<br>Age ______	Y □ N □<br>Unk □<br>Age ______	Y □ ________________________________<br>N □ Unk □<br>Age ______
Sibling 4<br>N/A □	Y □ N □<br>Unk □<br>Age ______	Y □ N □<br>Unk □<br>Age ______	Y □ N □<br>Unk □<br>Age ______	Y □ N □<br>Unk □<br>Age ______	Y □ N □<br>Unk □<br>Age ______	Y □ ________________________________<br>N □ Unk □<br>Age ______

MEDICAL HISTORY – Please provide the following information and give details where possible.

Has the participant ever been in hospital?

No ☐

Yes ☐ - please provide details below

Reason for hospital visit/admission	Dates
e.g., Bike accident – fractured left wrist	Aug 2010

Is the participant taking any **prescription medications**?

No, the participant is not taking any prescription medications ☐

Yes ☐ - please provide details below

Name of medication	Dose	How many doses are taken at the following times?
e.g., Ventolin	100mg	_1_ morning _0_ noon _0_ afternoon _0_ night ☐as needed
		___morning ___noon ___afternoon __night ☐as needed
		___morning ___noon ___afternoon __night ☐as needed
		___morning ___noon ___afternoon __night ☐ as needed
		___morning ___noon ___afternoon __night ☐ as needed
		___morning ___noon ___afternoon __night ☐ as needed

Is the participant taking any **over-the-counter** medications?

No, the participant is not taking any over-the-counter medications ☐

Yes ☐ - please provide details below

Name of medication	Dose	How many doses are taken at the following times
e.g., Iron tablets	40mg	1 morning 0 noon 0 afternoon 0 night ☐ as needed
		__morning ___noon ___afternoon __night ☐ as needed
		__morning ___noon ___afternoon __night ☐ as needed
		__morning ___noon ___afternoon __night ☐ as needed
		__morning ___noon ___afternoon __night ☐ as needed
		__morning ___noon ___afternoon __night ☐ as needed

Is the participant **allergic (has a bad reaction)** to anything, for example; pollen, penicillin, soya? ☐ Y ☐ N

If yes please provide details below

Allergic to	What happens?
e.g., Pollen	Sore itchy eyes, runny nose

Has the participant ever suffered from any of the following conditions? Please tick 'Unknown' for cases when the information is not available.

Condition	
Acanthosis Nigricans (i.e., dark, thickened patches of skin)	Y ☐ N ☐ Unk☐
ADD/ADHA (i.e., problems paying attention, sitting still)	Y ☐ N ☐ Unk☐
Back problems (i.e., crooked back, back pain)	Y ☐ N ☐ Unk☐
Hearing problems	Y ☐ N ☐ Unk☐
High blood pressure	Y ☐ N ☐ Unk☐
High cholesterol	Y ☐ N ☐ Unk☐
Jaundice (i.e., yellow skin)	Y ☐ N ☐ Unk☐
Muscle and bone problems (i.e., weak muscles, joint pain)	Y ☐ N ☐ Unk☐
Obstructive sleep apnoea (i.e., stops breathing or struggles for breath when sleeping)	Y ☐ N ☐ Unk☐
Polycystic ovary syndrome (PCOS)	Y ☐ N ☐ Unk☐
Seizures (i.e., shaking fits)	Y ☐ N ☐ Unk☐
Skin problems (i.e., acne, flaking skin, rashes, hives)	Y ☐ N ☐ Unk☐
Snores when sleeping	Y ☐ N ☐ Unk☐
Depression	Y ☐ N ☐ Unk☐
Metabolic syndrome	Y ☐ N ☐ Unk☐
Pre-Diabetes	Y ☐ N ☐ Unk☐
Asthma	Y ☐ N ☐ Unk☐
Fatty Liver Disease	Y ☐ N ☐ Unk☐
Hirsutism (excessive hairiness in girls where you wouldn't normally expect it i.e. face, back and forearms)	Y ☐ N ☐ Unk☐
Cardiovascular disease	Y ☐ N ☐ Unk☐

<u>Social Economics</u> – Please provide the following information. Tick one option for each question.

**What is the highest level of education you have completed?**	
University or college or equivalent	☐
Intermediate between secondary level and university (e.g. technical training)	☐
Secondary School	☐
Primary School only (or less)	☐
**In what occupational capacity are you presently working or (if no longer working) in what occupation did you last work?**	
Laborer	☐
Self-employed	☐
Employee	☐
Civil Servant (including judges and professional soldiers)	☐
Other: trainees, students, conscripts, etc.	☐
Never worked	☐
Unemployed	☐

PERI-NATAL HISTORY – Please provide the following information. Tick 'Unk' for cases when the information is not available.

What was the participant's birth weight?	☐☐.☐lbs.<br>OR<br>☐.☐kg
What was the participant's gestational period?	Weeks<br>Unk □
How many pregnancies has the biological mother had?	Unk □
Which pregnancy was the participant?	Unk □
Has the biological mother had gestational diabetes (GDM)?	Y □ N □ Unk □
If **YES**, during how many of her pregnancies did she had GDM?	
Did she have GDM when pregnant with the participant?	Y □ N □ Unk □
Did she breastfeed the participant?	Y □ N □ Unk □
If **YES**, for how long did she breastfeed the participant?	Weeks Months<br>Unk □
Was the participant conceived by IVF?	Y □ N □ Unk □
Is the participant a twin?	Y □ N □ Unk □
Did the biological mother smoke during her pregnancy with the participant?	Y □ N □ Unk □
Did the biological father smoke during the gestational period of the participant?	Y □ N □ Unk □
What age was the biological mother when pregnant with the participant?	Years

ANTHROPMETRIC MEASURES – Please record the following information.

Height	□□□.□cm
Weight	□□□.□kg
Waist circumference	□□□.□cm
Neck circumference	□□.□cm
Upper arm circumference	□□.□cm
Body fat	□□.□%
Fat-free mass	□□□.□kg
Muscle mass	□□□.□kg
Visceral rating	□□

BLOOD PRESSURE – Please record the following information.

Blood pressure 1	□□□ systolic	□□□ diastolic	mmHg	Heart rate 1	□□□	bpm
Blood pressure 2	□□□ systolic	□□□ diastolic	mmHg	Heart rate 2	□□□	bpm
Blood pressure 3	□□□ systolic	□□□ diastolic	mmHg	Heart rate 3	□□□	bpm

Biochemistry – Please provide the following information.

Time taken HH:MM

Is the participant fasting?

No ☐

Yes ☐

HbA1c	Y ☐ N ☐	☐☐☐mmol/mol	☐☐.☐%
Glucose	Y ☐ N ☐	☐☐.☐mmol/L	
Triglycerides	Y ☐ N ☐	☐☐.☐mmol/L	
Total cholesterol	Y ☐ N ☐	☐☐.☐mmol/L	
HDL-c	Y ☐ N ☐	☐☐.☐mmol/L	
LDL-c (calculated)	Y ☐ N ☐	☐☐.☐mmol/L	

ADDITIONAL COMMENTS – please use the space below to record any additional important or clinically significant information. Please document the reason why any measurements have been missed or omitted.

Activity	Completed by (Please initial)
**Ethnicity**	
**Family History**	
**Medical History**	
**Social economics**	
**Peri-Natal History**	
**Anthropometrics**	
**Blood Pressure**	
**Biochemistry**	

BLANK

Additional Questions – For completion by HCP – Associated Risk factors

Section 1: Overweight & Sedentary

Is your child's body mass index (BMI) above the 85th percentile? **Yes** **No**

Please use the charts and instructions provided to answer this question

Does your child watch TV/play computer games for more than 2 hours a day? **Yes** **No**

Section 2: Other risk factors

Look at the table below and find your child's age and gender. Measure their waist circumference (using the guide below). Is the result higher than the measurement given in the table? **Yes** **No**
For example if your son is 12 and his waist measurement is 85 cm please answer YES.

Gender	Age	Measurement (cm)
**Male**	12	84.5
	13	87.9
	14	91.3
**Female**	12	81.2
	13	84.1
	14	86.9

Has your child been diagnosed with any of the following?
Acanthosis Nigricans **Yes** **No**
(Dark patchy skin in places such as on the neck, groin or underarm)
Pre-diabetes **Yes** **No**
Fatty liver disease **Yes** **No**
Polycystic ovary syndrome **Yes** **No**

Has your child been told by they have any three of the factors below or that they have metabolic syndrome? **Yes** **No**

- High blood pressure
- High cholesterol
- Low HDL cholesterol
- High triglycerides
- High blood glucose levels, but not diagnosed with diabetes

Does your child have a first family history of diabetes? This includes only close blood relatives such as mother, father, sister or brother. **Yes** **No**

Is your child of non-white ethnicity? **Yes** **No**

Did your child gain more 2 lb (908 grams) a month between 0-4 months old? **Yes** **No**

Does your child have a high sugar intake?
For example more than 1.5 cans (or 532mls) of fizzy pop/ fruit juice a day? **Yes** **No**

"Small for gestational age". Please look at the tables bellow. Think back to how many weeks was your child when she or he was born and the weight that she or he had.

After looking the tables please answer to the question:

Did your child (girl or boy) have a weight under the weight corresponding to the week pregnant?

Yes **No**

**Girls**		**Boys**	
Number of weeks pregnant	Babies weight in grams	Number of weeks pregnant	Babies weight in grams
26 weeks	580,12	26 weeks	582,81
27 weeks	660,9	27 weeks	642,83
28 weeks	688,98	28 weeks	682,19
29 weeks	695,08	29 weeks	844,23
30 weeks	838,53	30 weeks	972,44
31 weeks	966,26	31 weeks	1115,43
32 weeks	1001,8	32 weeks	1182,73
33 weeks	1304,49	33 weeks	1399,56
34 weeks	1544,72	34 weeks	1605,18
35 weeks	1643,28	35 weeks	1738,47
36 weeks	1736,46	36 weeks	1937,52
37 weeks	1976,26	37 weeks	2019,78
38 weeks	2229,42	38 weeks	2339,03
39 weeks	2440,84	39 weeks	2506,71
40 weeks	2535,3	40 weeks	2601,94
41 weeks	2546,37	41 weeks	2678,09
42 weeks	2702,72	42 weeks	2746,55

Did your child weigh less than the weight given for the number of weeks you/their biological mother was pregnant for (i.e. were they small for their gestational age?)? **Yes** **No**

i.e. if your child was carried for 32 weeks and their birth weight was less than 3.75 lb (1702 grams) you would answer yes

Was your child never feed on breast milk? **Yes** **No**

Is either parent/guardian obese? Defined as having a BMI greater than 30kg/m^2 if White European or 27kg/m^2 for other ethnicity which can be calculated using the BMI calculator included?

Yes **No**

Please see BMI calculator
