# Supplementary material for: Evaluation and refinement of the PRESTARt tool for identifying 12–14 year olds at high lifetime risk of developing type 2 diabetes compared to a clinicians assessment of risk: a cross-sectional study
Source: BMC Endocr Disord. 2019 Jul 25;19:79. doi: 10.1186/s12902-019-0410-3 (PMC6659313; doi:10.1186/s12902-019-0410-3)
Supplement: Supplementary file 2 — This file includes an example (female participant version) of the questionnaire completed by all participants. (PDF 400 kb) [file 12902_2019_410_MOESM2_ESM.pdf]

# PHASE 1

# QUESTIONNAIRE

# FEMALE

# <u>Physical Activity</u>

**Physical activity** is any activity that increases your heart rate and makes you get out of breath some of the time.

**Physical activity** can be done in sports, playing with friends, or walking to school.

Some examples of **physical activity** are running, brisk walking, rollerblading, biking, dancing, skateboarding, swimming, soccer, basketball, football, & surfing.

**1a. Over the <u>past 7 days</u> on how many days were you physically active for a total of <u>60 mins</u> per day (don't include your PE class)? Please tick ONE box.**

| 0 days | 1 day | 2 days | 3 days | 4 days | 5 days | 6 days | 7 days |
|---|---|---|---|---|---|---|---|
| | | | | | | | |

**1b. Over a typical or usual week, on how many days are you physically active for a total of at least <u>60 mins</u> per day? Please tick ONE box.**

| 0 days | 1 day | 2 days | 3 days | 4 days | 5 days | 6 days | 7 days |
|---|---|---|---|---|---|---|---|
| | | | | | | | |

Continued on next page/…

# Sitting Activity

**2a. Think about a normal school week and write down how long you spend sitting down doing the following activities before and after school each day. If you do more than one of the activities at once (e.g. watching TV and using the internet), please pick the one that you are concentrating on the most.**

| Activity | Monday | | Tuesday | | Wednesday | | Thursday | | Friday | |
|---|---|---|---|---|---|---|---|---|---|---|
| | Hours | Mins | Hours | Mins | Hours | Mins | Hours | Mins | Hours | Mins |
| Watching a programme/film on TV/DVD/tablet/laptop | | | | | | | | | | |
| Using the computer for fun (laptop or games console) | | | | | | | | | | |
| Using a mobile phone to play games/search the internet/message friends | | | | | | | | | | |
| Using the computer/laptop for homework | | | | | | | | | | |
| Doing homework not on the computer/laptop | | | | | | | | | | |
| Reading for fun | | | | | | | | | | |
| Travel (car/bus/train) | | | | | | | | | | |
| Doing crafts/hobbies | | | | | | | | | | |
| Sitting around (chatting with friends/on the phone/chilling) | | | | | | | | | | |
| Playing/practising a musical instrument | | | | | | | | | | |

| | | | | | | | | | | |
|---|---|---|---|---|---|---|---|---|---|---|
| Other (please state) | | | | | | | | | | |

**2b. Think about a normal weekend and write down how long you spend sitting down doing the following activities. If you do more than one of the activities at once (e.g. watching TV and using the internet), please pick the one that you are concentrating on the most.**

| Activity | Saturday | | Sunday | |
|---|---|---|---|---|
| | Hours | Mins | Hours | Mins |
| Watching a programme/film on TV/DVD/tablet/laptop | | | | |
| Using the computer for fun (laptop or games console) | | | | |
| Using a mobile phone to play games/search the internet/message friends | | | | |
| Using the computer/laptop for homework | | | | |
| Doing homework not on the computer/laptop | | | | |
| Reading for fun | | | | |
| Travel (car/bus/train) | | | | |
| Doing crafts/hobbies | | | | |
| Sitting around (chatting with friends/on the phone/chilling) | | | | |
| Playing/practising a musical instrument | | | | |
| Other (please state) | | | | |

# What I eat

**3. How often do you usually have breakfast (more than a glass of milk or fruit juice)? Tick ONE box for weekdays and ONE box for the weekend.**

| Weekdays | | Weekends | |
|---|---|---|---|
| | I never have breakfast | | I never have breakfast |
| | One day | | I usually have breakfast on only ONE day of the weekend |
| | Two days | | I usually have breakfast on BOTH weekend days |
| | Three days | | |
| | Four days | | |
| | Five days | | |

**4a. During the past week, how often did you eat sweet or savoury snacks (e.g. crisps, chocolates, pasties, cakes, sweets, samosas) between main meals? Please tick ONE box.**

| 0 days | 1 day | 2 days | 3 days | 4 days | 5 days | 6 days | 7 days |
|---|---|---|---|---|---|---|---|
| | | | | | | | |

**4b. During the past week, how many snacks did you typically eat each day? Please tick ONE box.**

| None | 1 | 2 | 3 | 4 | 5 or more |
|---|---|---|---|---|---|
| | | | | | |

**5a. How many servings of vegetables do you usually eat EACH day? Please tick just ONE box.**

| I don't eat vegetables | 1 | 2-3 | 4-5 | 6 or more |
|---|---|---|---|---|
| | | | | |

**5b. How many servings of fruit do you usually eat EACH day? Please tick just ONE box.**

| I don't eat fruit | 1 | 2-3 | 4-5 | 6 or more |
|---|---|---|---|---|
| | | | | |

**6. How many times a week do you usually drink sugary drinks (e.g. fizzy drinks/pop (not including low-fat or diet versions), fruit juices, sports drinks, flavoured water (e.g. Ribena, Oasis)? Please tick just ONE box.**

| Never | Less than once a week | Once a week | 2-4 days a week | 5-6 days a week | Every day, once a day | Every day, more than once |
|---|---|---|---|---|---|---|
| | | | | | | |

Continued on next page/…

# My Body

**7a. Stages of Breast Development in Girls**

*Please indicate by ticking the box which set of pictures best describes you.*

☐ 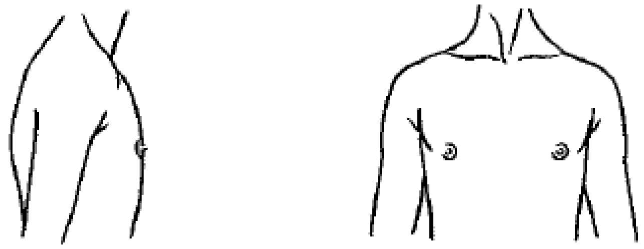

☐ 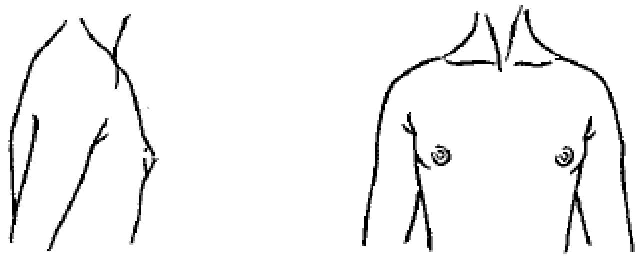

☐ 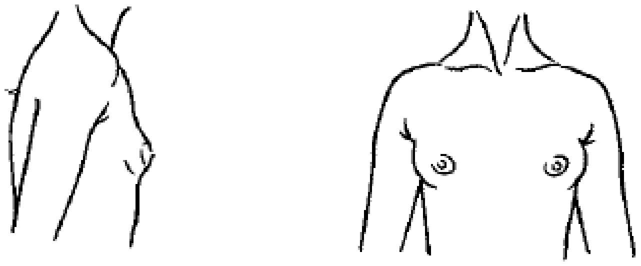

☐ 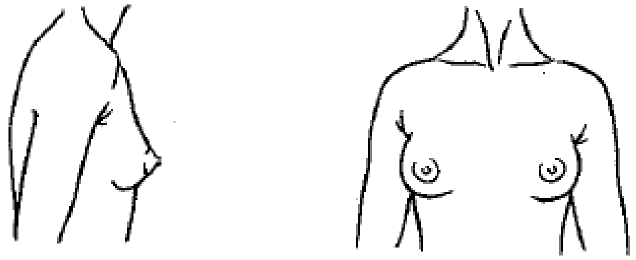

☐ 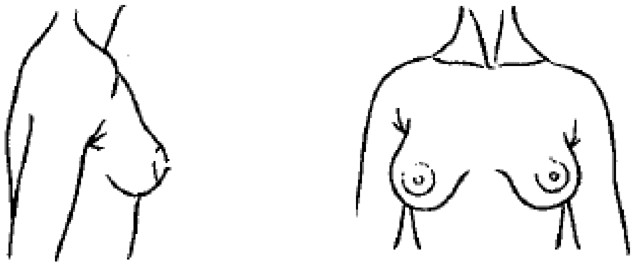

**7b. Stages of <u>Pubic Hair</u> Development in Girls**

*Please indicate by ticking the box which picture best describes you.*

☐ 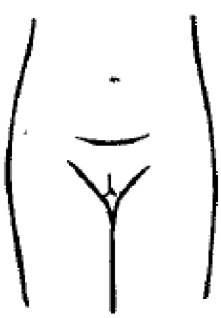 ☐ 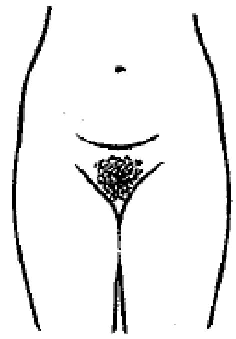

☐ 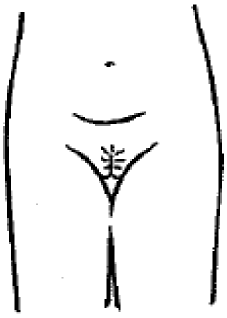 ☐ 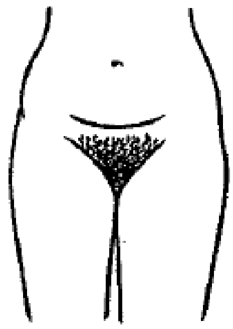

☐ 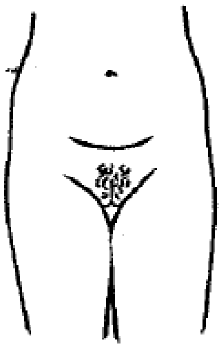 ☐ 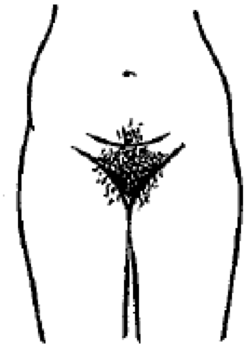

**Thank you for completing this questionnaire**
