# Supplementary material for: Evaluation and refinement of the PRESTARt tool for identifying 12–14 year olds at high lifetime risk of developing type 2 diabetes compared to a clinicians assessment of risk: a cross-sectional study
Source: BMC Endocr Disord. 2019 Jul 25;19:79. doi: 10.1186/s12902-019-0410-3 (PMC6659313; doi:10.1186/s12902-019-0410-3)
Supplement: Supplementary file 3 — Table S1. Family medical history summary statistics. Data reported as n (%). Table S2. Participant medical history summary statistics. Data reported as n (%) unless otherwise stated. Table S3. Socio-economic summary statistics. Data reported as n (%). Table S4. Perinatal history summary statistics. Data reported as mean (sd) unless otherwise stated. Table S5. Summary statistics for participant’s physical activity and sedentary behaviour. Data reported as n (%) unless otherwise stated. Table S6. Summary statistics for participant’s diet. Data reported as mean (sd). Table S7. Tanner stage summary statistics. Data reported as n (%). (DOCX 29 kb) [file 12902_2019_410_MOESM3_ESM.docx]

**SUPPLEMENTARY TABLES**

Table S1. Family medical history summary statistics. Data reported as n (%).

|  | UK  81 (12.7) | Germany  100 (15.7) | Portugal  226 (35.5) | Spain  129 (20.3) | Greece  100 (15.7) | Total  636 |
| --- | --- | --- | --- | --- | --- | --- |
| Number recruited |  |  |  |  |  |  |
| 1^st^ Degree Family History |  |  |  |  |  |  |
| Heart Disease^1^ | 1 (1.23) | 6 (6.0) | 1 (0.44) | 2 (1.55) | 10 (10.0) | 20 (3.1) |
| High Cholesterol^2^ | 22 (27.2) | 12 (12.0) | 27 (12.0) | 36 (27.9) | 34 (34.0) | 131 (20.6) |
| Cardiovascular Disease^3^ | 7(8.6) | 18(18.0) | 25(11.1) | 5(3.9) | 26(26.0) | 81 (12.7) |
| Type 1 Diabetes^4^ | 2 (2.5) | 1 (1.0) | 0 (0.0) | 1 (0.8) | 2 (2.0) | 6 (0.9) |
| Type 2 Diabetes^5^ | 15 (18.5) | 7 (7.0) | 14 (6.2) | 12 (9.3) | 15 (15) | 63 (9.9) |
| 2^nd^ Degree Family History |  |  |  |  |  |  |
| Heart Disease^6^ | 34 (42.0) | 39 (39.0) | 12 (5.3) | 56 (43.4) | 62 (62.0) | 203 (31.9) |
| High Cholesterol^7^ | 46 (56.8) | 36 (36.0) | 24 (10.6) | 71 (55.0) | 50 (50.0) | 227 (35.7) |
| Cardiovascular Disease^8^ | 45 (55.6) | 57 (57.0) | 23 (10.2) | 41 (31.8) | 69 (69.0) | 235 (36.9) |
| Type 1 Diabetes^9^ | 2 (2.5) | 0 (0.0) | 0 (0.0) | 4 (3.1) | 2 (2.0) | 8 (1.3) |
| Type 2 Diabetes^10^ | 45 (55.6) | 46 (46.0) | 68 (30.1) | 51 (39.5) | 43 (43.0) | 253 (39.8) |
| ^1^126 missing values, ^2^132 missing values, ^3^543 missing values, ^4^129 missing values, ^5^133 missing values, ^6^113 missing values, ^7^127 missing values, ^8^121 missing values, ^9^119 missing values, ^10^118 missing values. | | | | | | |

Table S2. Participant medical history summary statistics. Data reported as n (%) unless otherwise stated.

|  | UK | Germany | Portugal | Spain | Greece | Total |
| --- | --- | --- | --- | --- | --- | --- |
| Number recruited | 81 (12.7) | 100 (15.7) | 226 (35.5) | 129 (20.3) | 100 (15.7) | 636 |
| Previous Hospitalisation | 50 (61.7) | 54 (54.0) | 67 (29.7) | - | 63 (63.0) | 234 (36.8) |
| Number of times^1^, mean (SD) | 1.1 (1.1) | 0.9 (1.1) | 0.3 (0.6) | - | 1.1 (1.2) | 0.7 (1.0) |
| Prescription medications | 22 (27.2) | 24 (24.0) | 30 (13.3) | 23 (17.8) | 14 (14.0) | 113 (17.8) |
| Number of medications^1^, mean (SD) | 0.5 (0.9) | 0.4 (0.8) | 0.2 (0.6) | 0.3 (0.7) | 0.1 (0.6) | 0.1 (0.3) |
| OTC medications | 14 (17.3) | 14 (14.0) | 0 (0.0) | 0 (0.0) | 10 (10.0) | 38 (5.9) |
| Number of OTC medications^1^, mean (SD) | 0.2 (0.5) | 0.2 (0.5) | 0 (0.0) | 0 (0.0) | 0.1 (0.3) | 0.1 (0.3) |
| Allergies | 21 (25.9) | 31 (31.0) | 69 (30.5) | 30 (23.3) | 29 (29.0) | 180 (28.3) |
| Number of allergic reactions^1^, mean (SD) | 0.4 (1.0) | 0.7 (1.7) | 0.4 (0.8) | 0.3 (0.6) | 0.4 (0.5) | 0.4 (1.0) |
| Acanthosis nigricans | 1 (1.2) | 16 (16.0) | 0 (0.0) | 3 (2.3) | 10 (10.0) | 30 (4.7) |
| ADD/ADHD | 5 (6.2) | 5 (5.0) | 9 (4.0) | 5 (3.9) | 9 (9.0) | 33 (5.2) |
| Back Problems | 3 (3.7) | 32 (32.0) | 10 (4.4) | 5 (3.9) | 6 (6.0) | 56 (8.8) |
| Hearing Problems | 4 (4.9) | 4 (4.0) | 3 (1.3) | 0 (0.0) | 3 (3.0) | 14 (2.2) |
| High Blood Pressure | 0 (0.0) | 15 (15.0) | 0 (0.0) | 1 (0.8) | 7 (7.0) | 23 (3.6) |
| High Cholesterol | 1 (1.2) | 10 (10.0) | 3 (1.3) | 4 (3.1) | 6 (6.0) | 24 (3.8) |
| Jaundice | 5 (6.2) | 0 (0.0) | 0 (0.0) | 1 (0.8) | 2 (2.0) | 8 (1.3) |
| Muscle and bone problems | 9 (11.1) | 12( 12.0) | 1 (0.4) | 0 (0.0) | 5 (5.0) | 27 (4.3) |
| Obstructive sleep apnoea | 0 (0.0) | 16 (16.0) | 0 (0.0) | 1 (0.8) | 1 (1.0) | 18 (2.8) |
| Polycystic ovary syndrome^2^ | 0 (0.0) | 1 (2.0) | 0 (0.0) | 1 (1.5) | 0 (0.0) | 2 (0.7) |
| Seizures | 2 (2.5) | 1 (1.0) | 1 (0.4) | 1 (0.8) | 2 (2.0) | 7 (1.1) |
| Skin Problems | 23 (28.4) | 32 (32.0) | 7 (3.1) | 2 (1.6) | 29 (29.0) | 93 (14.6) |
| Snores when sleeping | 16 (19.8) | 25 (25.0) | 15 (6.6) | 1 (0.8) | 13 (13.0) | 70 (11.0) |
| Depression | 2 (2.5) | 5 (5.0) | 0 (0.0) | 1 (0.8) | 1 (1.0) | 9 (1.4) |
| Metabolic syndrome | 0 (0.0) | 19 (19.0) | 0 (0.0) | 0 (0.0) | 1 (1.0) | 20 (3.1) |
| Pre-diabetes | 0 (0.0) | 9 (9.0) | 0 (0.0) | 0 (0.0) | 0 (0.0) | 9 (1.4) |
| Asthma | 12 (14.8) | 10 (10.0) | 10 (4.4) | 29 (22.5) | 7 (7.0) | 68 (10.7) |
| Fatty Liver | 0 (0.0) | 7 (7.0) | 0 (0.0) | 1 (0.8) | 4 (4.0) | 12 (1.9) |
| Hirsutism | 1 (1.2) | 2 (2.0) | 0 (0.0) | 1 (0.8) | 3 (3.0) | 7 (1.1) |
| Cardiovascular disease | 0 (0.0) | 1 (1.0) | 0 (0.0) | 2 (1.6) | 1 (1.0) | 4 (0.6) |
| ^1^Mean (standard deviation)  ^2^Percentages calculated out of the total number of female participant in each site. | | | | | | |

Table S3: Socio-economic summary statistics. Data reported as n (%).

|  | UK | Germany | Portugal | Spain | Greece | Total |
| --- | --- | --- | --- | --- | --- | --- |
| Number recruited | 81 (12.7) | 100 (15.7) | 226 (35.5) | 129 (20.3) | 100 (15.7) | 636 |
| Highest education level^1^ |  |  |  |  |  |  |
| University / college / equivalent | 47 (58.0) | 29 (29.0) | - | 34 (26.4) | 37 (37.0) | 147 (23.1) |
| Intermediate between secondary and university | 17 (21.0) | 46 (46.0) | - | 55 (42.6) | 28 (28.0) | 146 (23.0) |
| Secondary school | 16 (19.8) | 2 (2.0) | - | 22 (17.1) | 25 (25.0) | 65 (10.2) |
| Primary school or less | 1 (1.2) | 23 (23.0)* | - | 16 (12.4) | 10 (10.0) | 50 (7.9) |
| Occupational capacity^2^ |  |  |  |  |  |  |
| Labourer | 4 (4.9) | 3 (3.0) | - | 1 (0.8) | 11 (11.0) | 19 (3.0) |
| Self-employed | 17 (21.0) | 9 (9.0) | - | 24 (18.6) | 22 (22.0) | 72 (11.3) |
| Employee | 47 (58.0) | 65 (65.0) | - | 60 (46.5) | 33 (33.0) | 205 (32.2) |
| Civil servant | 1 (1.2) | 3 (3.0) | - | 16 (12.4) | 20 (20.0) | 40 (6.3) |
| Other | 3 (3.7) | 8 (8.0) | - | 2 (1.6) | 1 (1.0) | 14 (2.2) |
| Unemployed | 8 (9.9) | 11 (11.0) | - | 24 (18.6) | 13 (13.0) | 56 (8.8) |
| Never worked | 1 (1.2) | 0 (0.0) | - | 0 (0.0) | 0 (0.0) | 1 (0.2) |
| ^1^228 missing values, ^2^229 missing values. | | | | | | |

*This percentage was clarified and it was established that the education levels in Germany are not equitable to the UK system with ‘Primary School’ being classed as school up to 16 years of age as opposed to 11 years of age in the UK. This may account for this unexpected difference.

Table S4. Perinatal history summary statistics. Data reported as mean (sd) unless otherwise stated.

|  |  | UK | Germany | Portugal | Spain | Greece | Total |
| --- | --- | --- | --- | --- | --- | --- | --- |
| Number recruited |  | 81 (12.7) | 100 (15.7) | 226 (35.5) | 129 (20.3) | 100 (15.7) | 636 |
| Birthweight (lbs)^1, 3^ | | 7.0 (1.3) | 7.4 (1.3) | - | 7.1 (1.4) | 7.0 (1.4) | 7.1 (1.4) |
| Gestational period (weeks)^4^ | | 39.1 (2.4) | 38.9 (2.0) | - | 38.9 (2.4) | 38.0 (2.0) | 38.7 (2.2) |
| Number of pregnancies^5^ | | 3.5 (1.6) | 3.0 (1.6) | - | 2.4 (1.1) | 2.4 (1.4) | 2.8 (1.5) |
| Pregnancy number, N(%)^5^ | 1 | 30 (37.0) | 51 (51.0) | - | 62 (48.1) | 55 (55.0) | 198 (31.1) |
|  | 2 | 22 (27.2) | 31 (31.0) | - | 46 (35.7) | 36 (36.0) | 135 (21.2) |
|  | 3 | 16 (19.8) | 7 (7.0) | - | 12 (9.3) | 6 (6.0) | 41 (6.5) |
|  | 4 | 5 (6.2) | 7 (7.0) | - | 4 (3.1) | 2 (2.0) | 18 (2.8) |
|  | $\geq$5 | 7 (8.6) | 4 (4.0) | - | 3 (2.3) | 1 (1.0) | 15 (2.4) |
| Gestational diabetes, n (%)^6^ | | 5 (6.2) | 14 (14.0) | - | 21 (16.3) | 12 (12.0) | 52 (8.2) |
| Number of pregnancies with Gestational diabetes, n (%)^7^ | 1 | 2 (40.0) | 12 (85.7) | - | 13 (61.9) | 11 (91.7) | 38 (73.1) |
|  | 2 | 0 (0.0) | 0 (0.0) | - | 5 (23.8) | 1 (8.3) | 6 (11.5) |
|  | 3 | 0 (0.0) | 1 (7.1) | - | 2 (9.5) | 0 (0.0) | 3 (5.8) |
|  | 4 | 1 (20.0) | 0 (0.0) | - | 1 (4.8) | 0 (0.0) | 2 (3.9) |
|  | 5 | 1 (20.0) | 0 (0.0) | - | 0 (0.0) | 0 (0.0) | 1 (1.9) |
| Gestational diabetes when pregnant with participant, n (%)^8^ | | 3 (3.7) | 6 (6.0) | - | 14 (10.9) | 5 (5.0) | 28 (4.4) |
| Breastfed, n (%)^9^ | | 63 (77.8) | 76 (76.0) | 131 (58.0) | 96 (74.4) | 78 (78.0) | 444 (69.8) |
| Breast feeding duration (weeks), median (IQR)^2^ | | 24 [11, 30] | 24 [12, 38] | - | 24 [16, 52] | 17.5 [7, 24] | 24 [12, 36] |
| IVF, n (%)^10^ | | 1 (1.2) | 1 (1.0) | - | 3 (2.3) | 5 (5.0) | 10 (1.6) |
| Twin, n (%)^11^ | | 4 (4.9) | 4 (4.0) | 6 (2.7) | 9 (7.0) | 7 (7.0) | 30 (4.7) |
| Mother smoking, n (%)^12^ | | 5 (6.2) | 23 (23.0) | - | 33 (25.6) | 21 (21.0) | 82 (12.9) |
| Father smoking, n (%)^13^ | | 20 (24.7) | 34 (34.0) | - | 60 (46.5) | 56 (56.0) | 170 (26.7) |
| Mother pregnancy age (years) | | 30.6 (5.5) | 28.7 (5.3) | - | 31.1 (5.4) | 30.6 (4.8) | 30.3 (5.3) |
| ^1^Birthweights recorded in Kg were transformed into pounds using the conversion rate 1Kg: 2.20462lbs.  ^2^Median duration and IQR presented due to the skew of the data.  ^3^238 missing values, ^4^237 missing values, ^5^229 missing values, ^6^125 missing values, ^7^2 missing values, ^8^582 missing values, ^9^74 missing values, ^10^229 missing values, ^11^4 missing values, ^12^227 missing values, ^13^233 missing values. | | | | | | | |

Table S5. Summary statistics for participant’s physical activity and sedentary behaviour. Data reported as n (%) unless otherwise stated.

|  |  | UK | Germany | Portugal | Spain | Greece | Total |
| --- | --- | --- | --- | --- | --- | --- | --- |
| Number recruited |  | 81 (12.7) | 100 (15.7) | 226 (35.5) | 129 (20.3) | 100 (15.7) | 636 |
| Number of days achieving 60 mins physical activity in the last week^1^ | | | | | | | |
|  | 0 days | 3 (3.7) | 6 (6.0) | 72 (31.9) | 14 (10.9) | 20 (20.0) | 115 (18.1) |
|  | 1 days | 12 (14.8) | 17 (17.0) | 36 (15.9) | 21 (16.3) | 8 (8.0) | 94 (14.8) |
|  | 2 days | 11 (13.6) | 21 (21.0) | 38 (16.8) | 22 (17.1) | 13 (11.0) | 105 (16.5) |
|  | 3 days | 15 (18.5) | 15 (15.0) | 20 (8.9) | 23 (17.8) | 27 (27.0) | 100 (15.7) |
|  | 4 days | 6 (7.4) | 20 (20.0) | 25 (11.1) | 23 (17.8) | 15 (15.0) | 89 (14.0) |
|  | 5 days | 10 (12.4) | 13 (13.0) | 15 (6.6) | 12 (9.3) | 3 (3.0) | 53 (8.3) |
|  | 6 days | 5 (6.2) | 2 (2.0) | 4 (1.8) | 7 (5.4) | 8 (8.0) | 26 (4.1) |
|  | 7 days | 17 (21.0) | 6 (6.0) | 16 (7.1) | 6 (4.7) | 6 (6.0) | 51 (8.0) |
|  | Median (IQR) | 3 (2,6) | 3 (2,4) | 2 (0,4) | 3 (1,4) | 3 (1,4) | 3 (1,4) |
| Number of days achieving 60 mins physical activity in a usual week^2^ | | | | | | | |
|  | 0 days | 5 (6.2) | 3 (3.0) | 35 (15.5) | 11 (8.5) | 13 (13.0) | 67 (10.6) |
|  | 1 days | 5 (6.2) | 14 (14.0) | 28 (12.4) | 12 (9.3) | 4 (4.0) | 63 (9.9) |
|  | 2 days | 4 (4.9) | 25 (25.0) | 62 (27.4) | 19 (14.7) | 19 (19.0) | 129 (20.3) |
|  | 3 days | 12 (14.8) | 22 (22.0) | 29 (12.8) | 33 (25.6) | 28 (28.0) | 124 (19.5) |
|  | 4 days | 18 (22.2) | 13 (13.0) | 32 (14.2) | 25 (19.4) | 11 (11.0) | 99 (15.6) |
|  | 5 days | 11 (13.6) | 14 (14.0) | 13 (5.8) | 18 (14.0) | 8 (8.0) | 64 (10.1) |
|  | 6 days | 11 (13.6) | 2 (2.0) | 12 (5.3) | 8 (6.2) | 11 (11.0) | 44 (6.9) |
|  | 7 days | 13 (16.1) | 7 (7.0) | 15 (6.6) | 2 (1.6) | 6 (6.0) | 43 (6.8) |
|  | Median (IQR) | 4 (3,6) | 3 (2,4) | 2 (1,4) | 3 (2,4) | 3 (2,4.8) | 3 (2,4) |
| Total hours spent sitting during weekdays | | | | | | | |
|  | Median (IQR) | 34.00 (27.36,40.86) | 33.83 (30.04,39.21) | 35.08 (31.46,37.62) | 21.00 (19.00,23.19) | 28.51 (25.12,31.89) | 30.04 (28.25,31.35) |
| Hours per day sitting during weekdays | | | | | | | |
|  | Median (IQR) | 6.8 (5.47,8.17) | 6.77 (6.01,7.84) | 7.02 (6.29,7.52) | 4.20 (3.80,4.64) | 5.70 (5.02,6.38) | 6.01 (5.65,6.27) |
| Total hours spent sitting during weekend | | | | | | | |
|  | Median (IQR) | 18.33 (15.43,21.96) | 17.92 (16.50,19.83) | 19.17 (16.73,22.31) | 13.33 (11.96,14.85) | 13.00 (11.97,16.00) | 16.29 (15.00,17.50) |
| Hours per day sitting during weekend | | | | | | | |
|  | Median (IQR) | 9.17 (7.72,10.98) | 8.96 (8.25,9.91) | 9.58 (8.37,11.15) | 6.67 (5.98,7.43) | 6.50 (5.99,8.00) | 8.15 (7.75,8.75) |
| ^1^3 missing values, ^2^3 missing values. | | | | | | | |

Table S6. Summary statistics for participant's diet. Data reported as mean (sd).

| Eating activity |  | UK | Germany | Portugal | Spain | Greece | Total |
| --- | --- | --- | --- | --- | --- | --- | --- |
| Weekdays -Breakfast^3^ | Never | 5 (6.2) | 6 (6.0) | 29 (12.8) | 15 (11.6) | 22 (22.0) | 77 (12.1) |
|  | One day | 3 (3.7) | 6 (6.0) | 2 (0.9) | 0 (0.0) | 4 (4.0) | 15 (2.4) |
|  | Two days | 1 (1.2) | 4 (4.0) | 11 (4.9) | 3 (2.3) | 6 (6.0) | 25 (3.9) |
|  | Three days | 6 (7.4) | 4 (4.0) | 9 (4.0) | 9 (7.0) | 7 (7.0) | 35 (5.5) |
|  | Four days | 7 (8.6) | 3 (3.0) | 7 (3.1) | 7 (5.4) | 6 (5.0) | 30 (4.7) |
|  | Five days | 57 (70.4) | 76 (76.0) | 167 (73.9) | 95 (73.6) | 52 (52.0) | 447 (70.3) |
| Previous week -snacks^4^ | Zero days | 1 (1.2) | 5 (5.0) | 22 (9.7) | 12 (9.3) | 9 (9.0) | 49 (7.7) |
|  | One day | 4 (4.9) | 10 (10.0) | 48 (21.2) | 32 (24.8) | 18 (18.0) | 112 (17.6) |
|  | Two days | 12 (14.8) | 25 (25.0) | 63 (27.9) | 47 (36.4) | 30 (30.0) | 177 (27.8) |
|  | Three days | 10 (12.4) | 18 (18.0) | 42 (18.6) | 12 (9.3) | 11 (11.0) | 92 (14.6) |
|  | Four days | 9 (11.1) | 9 (9.0) | 12 (5.3) | 11 (8.5) | 9 (9.0) | 50 (7.9) |
|  | Five days | 9 (11.1) | 10 (10.0) | 14 (6.2) | 5 (3.9) | 7 (7.0) | 45 (7.1) |
|  | Six days | 7 (8.6) | 4 (4.0) | 6 (2.7) | 2 (1.6) | 5 (5.0) | 24 (3.8) |
|  | Seven days | 27 (33.3) | 18 (18.0) | 19 (8.4) | 8 (6.2) | 10 (10.0) | 83 (13.1) |
| Snacks^2, 5^ | More than 5 | 14 (17.3) | 0 (0.0) | 68 (30.1) | 4 (3.1) | 5 (5.0) | 91 (14.3) |
| Vegetable servings^2, 6^ | 4+ servings | 13 (16.1) | 9 (9.0) | 65 (28.8) | 3 (2.3) | 18 (18.0) | 108 (17.0) |
| Fruit servings^2, 7^ | 4+ servings | 12 (14.8) | 13 (13.0) | 63 (27.9) | 10 (7.8) | 19 (19.0) | 117 (18.4) |
| 5-a-day^1^ |  | 41 (50.6) | 52 (52.0) | 129 (57.1) | 20 (15.5) | 41 (41.0) | 283(44.5) |
| Sugary Drinks^8^ | Never | 4 (4.9) | 10 (10.0) | 21 (9.3) | 17 (13.2) | 20 (20.0) | 72 (11.3) |
|  | Less than once a week | 11 (13.6) | 20 (20.0) | 31 (13.7) | 33 (25.6) | 29 (29.0) | 124 (19.5) |
|  | Once per week | 14 (17.3) | 18 (18.0) | 32 (14.2) | 39 (30.2) | 16 (16.0) | 119 (18.7) |
|  | 2-4 days per week | 28 (34.6) | 25 (25.0) | 62 (27.4) | 29 (22.5) | 21 (21.0) | 165 (25.9) |
|  | 5-6 days per week | 9 (11.1) | 5 (5.0) | 18 (8.0) | 5 (3.9) | 2 (2.0) | 39 (6.1) |
|  | Every day, once a day | 11 (13.6) | 9 (9.0) | 24 (10.6) | 3 (2.3) | 9 (9.0) | 56 (8.8) |
|  | Every day, more than once | 3 (3.7) | 12 (12.0) | 38 (16.8) | 2 (1.6) | 3 (3.0) | 58 (9.1) |
| ^1^The number of fruit and vegetables eaten by the participants per day is a rough estimate as the average value of the categories were taken to calculate the numbers.  ^2^per day.  ^3^7 missing values, ^4^3 missing values, ^5^2 missing values, ^6^4 missing values, ^7^4 missing values, ^8^3 missing values. | | | | | | | |

Table S7. Tanner stage summary statistics. Data reported as n (%).

|  |  | UK | Germany | Portugal | Spain | Greece | Total |
| --- | --- | --- | --- | --- | --- | --- | --- |
| Male, median (IQR) | Genitals | 3 (2, 4) | 3 (2, 4) | - | 3 (2, 3) | 3 (2.75, 4) | 3 (2, 4) |
|  | Pubic hair | 4 (2, 4) | 3 (2, 3) | - | 2 (2, 4) | 3.5 (3, 4) | 3 (2, 4) |
| Female, median (IQR) | Breasts | 4 (3, 4.5) | 3 (3, 4) | - | 4 (3, 5) | 4 (3, 4) | 4 (3, 4) |
|  | Pubic hair | 4 (3, 5) | 4 (3, 5) | - | 5 (4, 6) | 5 (4, 5) | 5 (3.5, 5) |
| Male external genitalia^1^ | Stage 1 | 6 (10.0) | 5 (10.0) | - | 4 (6.7) | 0 (0.0) | 15 (4.6) |
|  | Stage 2 | 18 (30.0) | 18 (36.0) | - | 20 (33.3) | 13 (24.1) | 69 (20.9) |
|  | Stage 3 | 10 (16.7) | 14 (28.0) | - | 21 (35.0) | 21 (38.9) | 66 (20.0) |
|  | Stage 4 | 12 (20.0) | 10 (20.0) | - | 8 (13.3) | 16 (29.6) | 46 (13.9) |
|  | Stage 5 | 10 (16.7) | 3 (6.0) | - | 5 (8.3) | 4 (7.4) | 22 (6.7) |
| Male pubic hair development^2^ | Stage 1 | 6 (10.0) | 4 (8.0) | - | 11 (18.3) | 0 (0.0) | 21 (6.4) |
|  | Stage 2 | 9 (15.0) | 16 (32.0) | - | 19 (31.7) | 11 (20.4) | 55 (16.7) |
|  | Stage 3 | 11 (18.3) | 18 (36.0) | - | 10 (16.7) | 16 (29.6) | 55 (16.7) |
|  | Stage 4 | 15 (25.0) | 7 (14.0) | - | 6 (10.0) | 16 (29.6) | 44 (13.3) |
|  | Stage 5 | 4 (6.7) | 2 (4.0) | - | 7 (11.7) | 9 (16.7) | 22 (6.7) |
|  | Stage 6 | 8 (13.3) | 1 (2.0) | - | 5 (8.3) | 2 (3.7) | 16 (4.9) |
| Female breast development^3^ | Stage 1 | 1 (4.8) | 1 (2.0) | - | 0 (0.0) | 1 (2.3) | 3 (1.0) |
|  | Stage 2 | 0 (0.0) | 8 (16.0) | - | 5 (7.3) | 3 (6.8) | 16 (5.3) |
|  | Stage 3 | 5 (23.8) | 18 (36.0) | - | 14 (20.3) | 16 (36.4) | 53 (17.4) |
|  | Stage 4 | 10 (47.6) | 15 (30.0) | - | 26 (37.7) | 19 (43.2) | 70 (23.0) |
|  | Stage 5 | 5 (23.8) | 8 (16.0) | - | 23 (33.3) | 5 (11.4) | 41 (13.5) |
| Female pubic hair development^4^ | Stage 1 | 0 (0.0) | 3 (6.0) | - | 2 (2.9) | 1 (2.3) | 6 (2.0) |
|  | Stage 2 | 2 (9.5) | 7 (14.0) | - | 3 (4.4) | 2 (4.6) | 14 (4.6) |
|  | Stage 3 | 4 (19.1) | 13 (26.0) | - | 4 (5.8) | 4 (9.1) | 25 (8.2) |
|  | Stage 4 | 8 (38.1) | 12 (24.0) | ` | 10 (14.5) | 10 (22.7) | 40 (13.2) |
|  | Stage 5 | 5 (23.8) | 10 (20.0) | - | 24 (34.8) | 21 (47.7) | 60 (19.7) |
|  | Stage 6 | 2 (9.5) | 4 (8.0) | - | 24 (34.8) | 6 (13.6) | 36 (11.8) |
| ^1^112 missing values, ^2^117 missing values, ^3^121 missing values, ^4^123 missing values. | | | | | | | |
