# Supplementary material for: Evaluation and refinement of the PRESTARt tool for identifying 12–14 year olds at high lifetime risk of developing type 2 diabetes compared to a clinicians assessment of risk: a cross-sectional study
Source: BMC Endocr Disord. 2019 Jul 25;19:79. doi: 10.1186/s12902-019-0410-3 (PMC6659313; doi:10.1186/s12902-019-0410-3)
Supplement: Supplementary file 4 — This file includes the final prestart tool. (DOCX 13 kb) [file 12902_2019_410_MOESM4_ESM.docx]

The refined final PRESTARt risk tool for identifying adolescents with a high lifetime risk of developing T2DM.

| A 12-14 year old inclusive will be considered to be at high lifetime risk of developing T2DM if they have a positive response to the question in Section 1 and to at least one question in Section 2.  Section 1   - Body mass index (BMI) above the 85th percentile   Section 2   - High waist circumference - Watch TV/play computer games for more than 2 hours a day - Do less than 60 minutes of physical activity a day - Eat less than 5 portions of fruit or vegetables a day - Have a family history of diabetes - Have a high sugar intake - Never fed on breast milk - Either parent/guardian obese |
| --- |
